# Quorum Sensing Inhibitors: Curbing Pathogenic Infections through Inhibition of Bacterial Communication

**DOI:** 10.22037/ijpr.2020.113470.14318

**Published:** 2021

**Authors:** Shaminder Singh, Sonam Bhatia

**Affiliations:** a *Regional Centre for Biotechnology, NCR Biotech Science Cluster, 3* ^*rd *^ *Milestone, Faridabad-Gurugram Expressway, Faridabad - 121 001, Haryana, India. *; b *Department of Pharmaceutical Science, SHALOM Institute of Health and Allied Sciences, Sam Higginbottom University of Agriculture, Technology and Sciences (SHUATS), Naini-211007, Prayagraj, Uttar Pradesh, India.*

**Keywords:** Quorum sensing inhibitors, Drug resistance, CviR and LasR receptors, Biofilm inhibition

## Abstract

Currently, most of the developed and developing countries are facing the problem of infectious diseases. The genius way of an exaggerated application of antibiotics led the infectious agents to respond by bringing a regime of persisters to resist antibiotics attacks prolonging their survival. Persisters have the dexterity to communicate among themself using signal molecules via the process of Quorum Sensing (QS), which regulates virulence gene expression and biofilms formation, making them more vulnerable to antibiotic attack. Our review aims at the different approaches applied in the ordeal to solve the riddle for QS inhibitors. QS inhibitors, their origin, structures and key interactions for QS inhibitory activity have been summarized. Solicitation of a potent QS inhibitor molecule would be beneficial, giving new life to the simplest antibiotics in adjuvant therapy.

## Introduction

Communication among bacteria using chemical signaling molecules is not a new concept but has been established long ago (1). An established intra- and inter-species cell-to-cell communication among bacteria had changed an age-long belief of their existence in planktonic forms without any communication between them. Bacterial species form colonies in which the behavior of an individual bacterial cell is governed by the release of small diffusible molecules called auto-inducers (AI). The number of bacterial cells increases with an increase in the extracellular concentration of autoinducer molecules (2, 3). When the concentration of autoinducer molecules had attained a particular threshold, then it triggers the binding of autoinducer molecules to their cognate receptors within the bacterial cell and thereby upregulating the expression of certain genes in synchrony. Such communication behavior has been termed as quorum sensing (QS) (4). 

**Figure 1:**
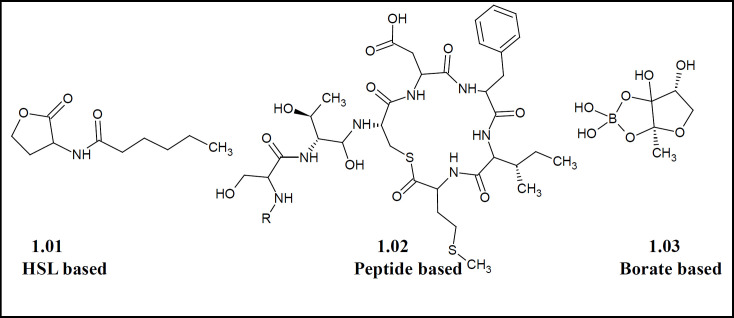
Structural representation of different classes of autoinducers that are involved in QS

The nature of signaling molecules responsible for such traits could be either acyl-homoserine lactones (**1.01**), small peptides (**1.02**) or complex molecules like borates derivatives (**1.03**), as shown in Figure 1. This communication among bacterial cells is responsible for various phenotypic traits like antibiotic production (5), antibiotic resistance (6), biofilm formation (7), bio-surfactant production (8), bioluminescence (9), conjugative DNA transfer (10) and virulence factor production (11).

**Mechanism of Quorum Sensing**

**Figure 2:**
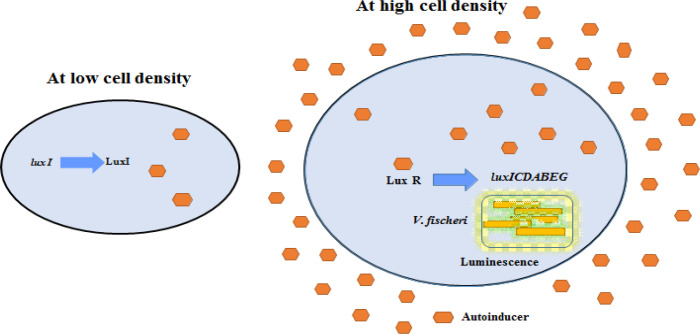
Schematic representation of QS mechanism in marine bacterium *V. fischeri*

The mechanism of QS was first discovered in the bioluminescent marine bacterium *Vibrio ﬁscheri (V. ﬁscheri).* Here a *luxI/luxR* based QS operates where the gene *luxI* encodes enzyme autoinducer synthase responsible for autoinducer production and another gene *luxR* mediate the production of receptor protein LuxR. N-acyl homoserine lactone (AHL) is produced in response to *luxI* gene expression at low microbial cell density. AI then diffuses to the surrounding medium increases its concentration; when the amount of AI reaches up to threshold levels, it binds to LuxR, forming a cytoplasmic AI-R complex that acts as DNA binding transcriptional activator. This complex activates (12) transcription of a *lux* operon (*luxCDABEG*), resulting in an increased cellular level transcription of messenger-RNA encoding for bioluminescence and synthesis of AI, as shown in Figure 2 (1, 13 and 14).

**Figure 3:**
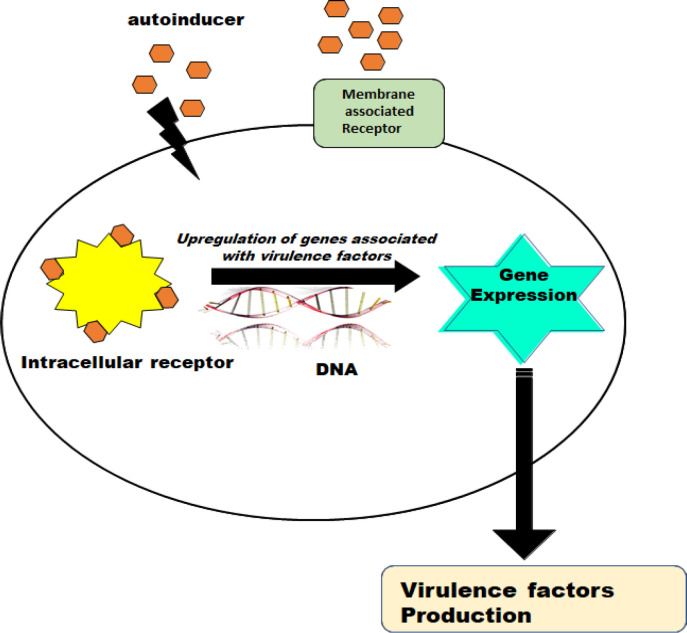
Mechanism depicting the production of virulence factors in the presence of autoinducer

On reaching threshold concentration, AI molecules direct the expression of their own as well as other virulence factors leading to the phenomenon of quorum sensing (QS) (Figure 3). These signaling molecules vary widely in their nature, such as (a) AHLs, (b) oligopeptides (5-10 amino acid), (c) Furanosyl borate (Autinducer-2, AI-2), (d) Hydroxyl-palmitic acid methyl ester and (e) Methyl dodecanoic acid (15, 16). 

**Figure 4:**
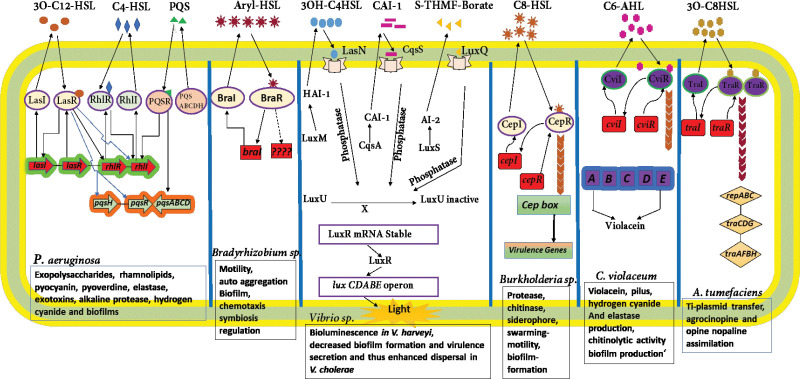
Summary of QS systems found in different Gram-negative Bacteria

Among the QS systems, the most widely studied QS AI molecules are those belonging to the class (a) AHL; these are produced mainly by Gram-negative bacteria, which binds to the regulatory proteins, (b) peptide; these are seen in Gram-positive bacteria and associated with membrane-bound receptor histidine kinase as shown in Figure 4 (17, 18).

**Autoinducers types**

*AHL based autoinducers*

The complexity of QS systems is highly conserved, and in most Gram-negative bacteria, AI molecules are produced by the activity of a synthase enzyme that utilizes S-adenosylmethionine along with an intermediate of fatty acid biosynthesis, *i.e*., acyl-acyl carrier protein. Enzyme synthase belongs to the LuxI family of AHL synthases, and the synthesized AI bind to their cognate receptors activating the transcription through the transcriptional regulator LuxR (19). In different Gram-negative bacteria, different LuxI homologs are responsible for the generation of different AHL, which usually differ in the length of the acyl side chain (C4-C18) and modification on the side acyl chain with substitution at position C3, which could be either carrying an oxo- or hydroxyl group or unmodified. 

This production and accumulation of AI account for characteristic patterns of QS-dependent phenotypic traits among bacterial populations like bioluminescence in* Vibrio ﬁscheri* (*V. ﬁscheri*), which rapidly shift at the quorum concentrations from an “off” state to an “on” state (20). Zhang *et al. *2002, in their studies, had reported that the binding of AI-LuxR on DNA fragments (promoter region of the gene) leads to the expression of genes regulated by QS systems. Various species of *Pseudomonas, Sinorhizobium *and *Vibrio *were found harboring multiple QS systems (21). 

In *Vibrio *sp., the LuxI-LuxR system regulates QS activity where LuxI is AI synthase enzyme regulating the production of N-(3-oxohexanoyl)- homoserine lactone (**1.04**) as shown in Table 1 (22). However, in the case of *Vibrio harveyi* *(V. harveyi),* the QS system operates *via* three different pathways known as HAI-1, CAI-1 and AI-2. HAI-1 is a chemical hydroxyl derivative of N-butanoyl homoserine lactone (**1.06**) regulating species-specific AHL pathway while CAI-1 is (S)-3-hydroxytridecan-4-one facilitating bacterial intergeneric communications and AI-2 pathway is responsible for interspecies communication where AI-2 chemically is a furanosyl borate diester (23). Greenberg and co-workers, 1994) reported initial signals of cross-species communication of QS by experimentally demonstrating *lux-*based QS in *Escherichia coli (E. coli). *Similarly, LasR receptor of *Pseudomonas *bound to its AI was found to activate *luxR *based bioluminescence in *E. coli*. This group also suggested that conserved autoinduction-related elements might be present within promoter regions of QS genes (24). 

In *Pseudomonas aeruginosa* *(P. aeruginosa), *three QS genes (*lasI*, *rhlI *and *qscI*) were reported to produce two AHL molecules viz. N-(3-Oxododecanoyl)-L-homoserine lactone (**1.05**) and N-butanoyl homoserine lactone (**1.06**), which further bind to their corresponding transcriptional receptors (LasR, RhlR and QscR) (25-27). In *Pseudomonas *species, involvement of signal molecule 2-heptyl-3-hydroxy-4-quinolone (PQS **1.16**, Table S1, in supplementary file) for connecting two QS systems was a key finding (4,28). In *P. aeruginosa, *a gene coding for a homolog of the signal receptors (LasR and RhlR) is also known to code for the receptor QscR and it mainly regulates repression of *lasI *(29). 

In *Agrobacterium* *tumefaciens (A.* *tumefaciens)* (plant pathogenic bacterium), virulence is regulated by TraI/TraR based QS system, causing tumor formation in the host plant through the transfer of oncogenic Ti plasmid. Synthase gene *traI *codes for the production of AI signal N-(3-oxoctanoyl)-homoserine lactone (**1.07**) (Table 1), combine with host signal and lead to conjugation of the pathogen (10,19,30,31). 

In *Chromobacterium violaceum (C. violaceum) *ATCC 31532, a number of multiple characteristics such as the production of purple pigment violacein, hydrogen cyanide and exoproteases are all regulated through CviI/CviR QS system (2). The endogenous AHL molecule N- hexanoyl-L-homoserine lactone (**1.08**) plays a major role in the expression of phenotypic traits in this pathogen (32). 

The occurrence of a VanI/VanR QS system in *Vibrio anguillarum (V. anguillarum)*. They found N-(3-oxodecanoyl)-L-homoserine lactone (**1.09) **as AI and cloned the synthase gene *vanI *responsible for AI synthesis. Further, they found that *vanI *mutant is also able to show virulence, indicating the presence of other QS systems (33). In the Gram-negative bacteria, *Burkholderia cepacia* (*B. cepacia*), the presence of CepR/I QS system in which AI molecule N-octanoyl-homoserine lactone (**1.10**) was found as a homolog of LuxR/LuxI system (34). This bacterium is usually associated in colonized form within the lungs of CFTR patients (35). 

In *B. mallei *species, the presence of the LuxI/R QS system is reported. In this organism, *BmaI1 *and *BmaI3 *are the synthase genes responsible for the production of signal molecules- octanoyl homoserine lactone (**1.11**) and N-3-hydroxy-octanoyl homoserine lactone (**1.12**) as shown in Table 1. These molecules interact with their cognate receptors *BmaR1 *and *BmaR3, *respectively (36,37). 

*Sinorhizobium meliloti (S. meliloti) *is a free-living soil bacterium found to be associated in a symbiotic relationship with a legume *Medicago sativa *(38)*. *This bacterium possesses three QS systems, namely (a) Sin, (b) Tra and (c) Mei. *SinI *and *SinR *genes were identified, representing effective QS systems that produce AHLs with alkyl chains having the number of carbons in a range of 12 to 18 (C12-HSL to C18-HSL) where compound **1.14** is N-palmitoyl-L-homoserine lactone (39). Table 1 enlists the structure of various autoinducers along with their respective gene and source of production.

*Quinolone based QS system*

**Figure 5:**
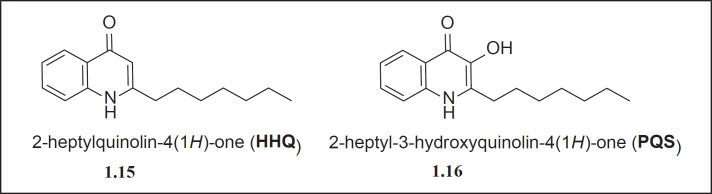
Structures of Quinolone based autoinducers

Along with the production of acyl-homoserine lactone as QS signals in *P. aeruginosa*, another class of autoinducers is 4-hydroxy 2-alkylquinolones (HAQs) and derivatives of 2-heptylquinolone (HHQ) (**1.15**), including hydroxy derivatives like PQS (**1.16**) (Figure 5). This QS system is responsible for the production of innate cytokines and thus helps the bacterial population to spread its pathogenicity, including its role in biofilm development (40).

*Peptide-based quorum sensing*

QS-induced gene expression is regulated in Gram-positive bacteria through oligopeptides (**1.02, **Figure 1). Gram-positive bacteria use two-component adaptive response proteins for sensing QS signals. As peptides are impermeable to biological membranes, their secretion is done through specialized transporters such as ATP-binding-cassette transporter, where they are post-translationally processed and secreted. The process of QS in *Bacillus subtilis* *(B. subtilis)*, *Streptococcus pneumoniae* *(S. pneumoniae) *and *Staphylococcus aureus* *(S. aureus) *was found to be under the control of peptide pheromones and two-component signal-transduction systems (41). 

In *B. subtilis*, the QS mechanism is controlled by the pheromone ComX and the ComP-ComA two-component systems (42). *Streptococcus mutans (S. mutans)*, also known to produce a quorum-sensing peptide called competence-stimulating peptide (CSP) pheromone under stress conditions and induces cell death in a fraction of the bacterial population in response to high levels of CSP pheromone (43).

*Furanosyl borate diester based QS*

This class of autoinducer is labeled as AI-2 and serves as a “universal signal,” which is produced mainly by both Gram-positive and Gram-negative bacteria in response to LuxS protein and gets involved in cross-species communication. The structural framework of AI-2 (**1.03**) is a novel furanosyl borate diester with no similarity to other autoinducers. The presence of a boron atom in AI-2 is especially intriguing because the functional role of boron in biological systems is not much explored. There is higher involvement of AI-2 in the QS circuit of bioluminescent bacterium *V. harveyi* (18).

**Biofilm formation in relation to quorum sensing**

The QS-mediated biofilm formation has been found responsible for increasing the pathogenicity of pathogens like *P. aeruginosa*, *Acinetobacter baumannii*, *Serratia marcescens (S. marcescens)* and *S. aureus *(44-47). 

The process of QS is responsible for bacterial biofilm formation in which the bacterial cells show altered physiological behavior leading to increased resistance towards adverse conditions, including antibiotic attack. The importance and involvement of membrane vesicles in the process of bacterial communication are well established (48).

This observation is further supported by the report of Mashburn and Whiteley, who mentioned the direct role of membrane vesicles in the transfer of QS signaling molecules, thereby initiating biofilm formation (49). The structural data of biofilm have been reported by Palsdottir *et al*., using electron tomography to describe the macromolecular ultrastructure of biofilm, displaying the community behavior of bacterial cells in biofilm instead of acting as individual identities (50). 

Biofilm-associated methicillin-resistant *S. aureus* (MRSA) infections are prone to clinical antibiotic failure. A highly reduced susceptibility of vancomycin was found in the presence of biofilm models (54). An enhancement in the therapeutic activity of amphotericin B against clinical isolates treated with QS inhibitor has been observed by inhibiting the biofilm formation in *Candida albicans (C. albicans) *(51). You *et al. *have shown the increased susceptibility of *Pseudomonas *biofilm towards the treatment with tobramycin and sodium dodecyl sulfate in the presence of QS inhibitor, and later on, the group led by Bjarnsholt *et al. *have demonstrated similar effects using garlic extract (52, 53). 

**QS mediated rhamnolipids production**

Rhamnolipids are hemolysins belonging to the class of glycolipids, specifically a key virulence determinant in *P. aeruginosa. *It is found to be the only virulence factor that is associated with the mortality of the patients on ventilator-pneumonia with lung and corneal infections (54). The role of Rhl QS systems that controls the genes critically involved in the regulation of the biosynthesis of rhamnolipids was also well established (55). The rhamnolipids production in the *P. aeruginosa *is under the regulation of QS mediated genes and could be quantified (56). 

**QS mediated exopolysaccharide production**

The conversion of *P. aeruginosa *to its mucoidal phenotype is regulated by the overproduction of alginate, an exopolysaccharide that forms a highly resistant complex-structured biofilm. The matrix plays a crucial role in the bioﬁlm resistant phenotype. In support of this, a study was carried out by Alkawash and co-workers showed that the biofilm of mucoidal *P. aeruginosa *was degraded in the presence of alginate lyase, which led to the enhanced antibiotics mediated killing (57).

In non-mucoidal forms of *P. aeruginosa *(PAO1 and PA14), the alginate is not the main component of the biofilm matrix and instead, the two loci, the *psl *(polysaccharide synthesis locus) in *P. aeruginosa *PAO1 and *pel *genes in *P. aeruginosa *PA14 (PAK) have been found to regulate the production of other polysaccharides and biofilm formation during *in-vitro *assays (58,59). This evidence clearly established a link between the expression of *psl *locus and QS in PAO1; however, in PA14, *psl *genes are known to be QS regulated. The quantitative measurement of the exopolysaccharide production can be utilized as a measure of bacterial pathogenesis and its inhibition can be utilized to measure the effectiveness of QS inhibitors (60). 

**QS mediated virulence gene expression**

**Figure 6:**
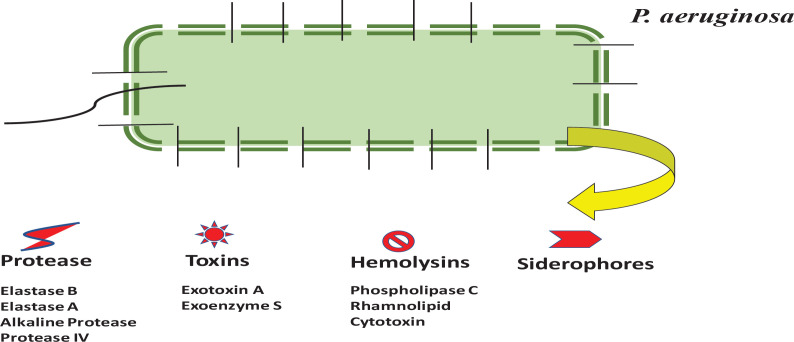
Pictorial representation of various virulence factors released from *P. aeruginosa*

QS mediates the expression of various virulence genes that are expressed in a concerted manner and culminates with an increase in virulence. The involvement of RhlR-RhlI based QS system in *P. aeruginosa *is responsible for the production of several virulence factors that make this pathogen to grow in high cell density during the condition of pneumonia was also well established (61). The various virulent factors which are responsible for the pathogenicity of *P. aeruginosa* are protease, exotoxin, phospholipase and siderophores, *etc*., (62-64), which are pictorially represented in Figure 6. 

Quorum-sensing proteins LuxO and HapR were found to regulate the virulence gene expression in *Vibrio cholerae* (65). The story of QS-mediated virulence expression is also seen with the plant pathogen *D. dadantii, *which secretes cell wall degrading enzymes responsible for causing soft-rot symptoms (66).

**Other QS system in various microorganisms**

Few other varied QS systems (homologs of LuxI/LuxR) were observed in species that do not synthesize their own signaling molecule, but they do respond to autoinducers produced by other organisms. In *E. coli, *a *sdiA*, a homolog of *luxR* QS system found to operate that causes changes in the gene expression and phenotypes in response to the exogenous AHLs (67). While pathogen *B. cepacia *response to signaling, molecules secreted by *P. aeruginosa *in response to its QS mechanism (68). The virulence factors released by the pathogen help in sustaining its survival. A dimorphic fungus, *C. albicans *found to produce farnesol (C15H26O) which is responsible for the QS-mediated pathogenic activity of this fungus (69). There are also reports of the existence of *luxR* homologs in certain classes of bacteria where the autoinduction phenomenon does not operate and some distinct exogenous molecules are binding with the receptor protein, namely ‘LuxR Solos’ (70).

**Inhibition of Quorum Sensing**

The protective mechanism of biofilm development in pathogenic bacteria through the process of QS helps bacterial cells to tolerate even higher concentrations of antibiotics and thus lead to the failure in their drug action. This insensitivity towards antibiotics is also among the prime cause of drug resistance (71). The discovery of “bacterial communication” with the help of chemical species has opened a new arena in the field of antimicrobials drug discovery. The process of virulence production is under the control of QS and thus, by inhibiting this phenomenon, the production of virulence factors can be decreased to a greater extent. Hence, blocking QS signaling is an effective tool to curb bacterial pathogenesis without any significant effect on bacterial growth and thereby reducing the risk of persisters development.

Different approaches are known which are used to inhibit the QS signaling viz. (a) antagonist of AHL, cause reduction in AHL activity, (b) degradation of AHL molecules, (c) inhibition of QS signals. Various synthetic leads and natural analogs as an antagonist of AHL were reported. Among popular possibilities, enzymatic degradation is also one of the most applied approaches. Kaufmann *et al. *2006 and Dembitsky *et al. *2011 suggested the use of antibodies and decoy receptors as a novel approach to inhibit QS signals (72, 73). 

**QS inhibitors**

*Synthetic inhibitors*

*Natural product-based inhibitors*

*Synthetic Inhibitors*

This section is divided into parts depending upon the origin of AHL-based QS inhibitor molecules.

*AHL based analogs*

*AHL Head group containing analogs*

A significant number of novel *N*-acyl homoserine lactone derivatives have been developed, which have AHL-based head groups. Their QS inhibition activity is confirmed on the basis of their ability to inhibit the violacein production in *C. violaceum *(CV12472) wild and CV026 mutant type strains. N-decanoyl-homoserine lactone derivative (**1.17**) and chlorolactone (CL) (**1.18**) (Table S1) are currently the most effective compounds to inhibit violacein production reported to date (74).

Blackwell and coworkers have synthesized a series of AHL analogs with improved catalytic yields by using a solid-phase synthetic strategy. The stereochemistry of compounds was chosen by keeping steric and binding site functionality in mind. Three AHL analogs (**1.19-1.21**) (Table S1) show significant LuxR-type protein activity against TraR in *A. tumefaciens *and LasR in *P. aeruginosa*. The structural features of ligands compromise of lactone head group, hydrophobic acyl groups and a linker. The presence of electron-withdrawing group (-Br (**1.21**) and -CF_3 _(**1.22**)) at the hydrophobic region increases the activity of these compounds as Compounds **1.19 **and **1.21 **also found to inhibit biofilm formation in *P. aeruginosa *at 50 µM concentration (75, 76). 

The synthetic analogs of *N*-acyl-homoserine lactone have been evaluated against the production of bioluminescence in *V. fischeri*. The acyl chain was fixed to four carbons long, and the phenyl substituted analogs (**1.23 **and **1.24**, Table S1) was found to have antagonistic activity (77).

Subsequently, the replacement of the carboxamide bond of homoserine lactone with its sulfonamide bioisostere (**1.25**) leads to the formation of a potent inhibitor that prevents the formation of active LuxR dimer and thus prevent bacterial QS signaling (78).

On similar lines, the synthesis of a novel series of *N*-sulfonyl homoserine lactone was done and found that the compounds with an *ortho *substitution to sulfonyl group have shown excellent QS inhibition activity with an IC50 value in the range of 1.66 – 4.91 µM for violacein production (79). In 2005, Persson *et al. *synthesized several analogs of AHL carrying sulfur atoms in the side chain. The derivatives have shown positive outcomes in the form of potent inhibitors of the LuxR and LasR/RhlR-based QS systems. Derivatives belonging to the N-acyl-3-amino-5*H*-furanone class along with their C4-substituted halogen analogs, were found to show promising activity against the LuxR-dependent bacterial QS system. Both types of analogs proved to be QS inhibitors, the halogenated compounds being significantly more active (80). 

*The non-AHL head group containing analogs*

In the design of QS inhibitors, the scientific community also considered some molecules which are mimics of natural acyl-homoserine lactone but lacks AHL head group. Suga and co-workers, 2003 have discovered a new antagonist, 3-oxo-C12-(2-aminocyclohexanol) (**1.26**), which activates the LasR receptor. They hypothesized that a 5-or 6-membered ring with a keto or hydroxyl group adjacent to amine is sufficient to show binding with LasR (81).

QS in Gram-negative bacteria *Burkhold-eria glumae *is controlled by binding N-octanoyl-L-homoserine lactone to its cognate receptor, TofR. An inhibitor (J8-C8) (**1.27**) of acyl-homoserine lactone synthase (TofI) which can inhibit the formation of N-octanoyl-L-homoserine lactone at ∼35 μM, has been identified. X-ray crystallographic characterization revealed the stabilization of the binding site segment Gly32–Glu40 by the inhibitor compared to its disordered geometry in the apo form (82). 

The use of thiolactone derivatives (**1.28**) with the potential to inhibit the autoinducer of *P. aeruginosa *QS(AHL-2) was reported (83). The authors have developed the conjugates of antibiotic-AHL analogs by linking them covalently. The efficacy of this system in disrupting nascent and mature biofilms was found better than the free antibiotic.

Similar analogs were also reported by McInnis and Blackwell, which can act by disturbing the interaction between AHL and LasR and thus prevent biofilm formation and virulence production in bacterial pathogens (84). Compounds belonging to the thiolactone class can remain active for a longer period of time in comparison to their natural lactone derivatives. 

The synthesis of secondary metabolite derivatives of *Delisea pulchra (D. pulchra) *which are capable of interfering with AHL-mediated QS in *P. aeruginosa, *was also reported*. *These compounds are characterized by an exocyclic double bond with bromine substitution. Both the compounds (**1.29**) and their related congener (**1.30**) (Table S1) have also shown efficacy against *P. aeruginosa *pathogenicity in lung infections in the mouse model (85).

The enamide group in these molecules helps in specific binding in the target protein by inducing preferential conformational changes in these molecules. In addition, the presence of the halogen atom could enhance the fit of the lactone ring through specific interactions with strictly conserved residues in the LuxR protein family, Asp79, Trp94 and Ile81 (86).

The compounds belonging to the furanone class have also been isolated from the *Penicillium sp*. This filamentous fungus produces secondary metabolites in response to its chemical defense mechanism; two chemical species identified as penicillic acid (**1.31**) and patulin (**1.32**, Table S1) show QS inhibitory activity (87). The identification of the inhibitors capable of interfering with the QS-controlled violacein production in *C. violaceum *by screening a library of compounds based on furanone class that is isolated from *S. antibioticus *and several synthetic analogs thereof also reported (88).

*Non-AHL based analogs*

Several attempts had been made in this field for developing analogs that are structurally distinct from native AHLs. Major exploited areas are the screening of natural products and chemically synthesized compounds. 

An ultra-high throughput cell-based assay was developed to screen a library of ∼20,000 compounds against LasR-dependent gene expression. The most active compounds V-06-018 (**1.33**) and PD12 (**1.34**) (Table S2, in supplementary file) are having 12 carbon aliphatic side chains similar to that of native autoinducer 3-oxo-N-dodecyl- L-homoserine lactone, with the non-AHL core of phenyl and tetrazole rings. The compounds act as inhibitors of LasR and down-regulate the gene expression (89).

In this quest, a number of compounds belonging to various classes viz. para-benzoquinone (**1.35**), 2,4,5-tri-bromo-imida-zole (**1.36**), indole (**1.37**), 3-nitro- benzene-sulfonamide (**1.38**) and 4-Nitro-pyridine-N-oxide (4-NPO) (**1.39**) have been identified (Table S2). Through DNA microarray-based analysis, it was found that 4-NPO is the most effective inhibitor and is capable of down-regulating 37% of the QS-regulated genes in *P. aeruginosa *(90).

The cyclic dipeptide moieties isolated from the cultures of various bacterial species have also shown activity against bacterial quorum sensing. The chemically isolated dipeptide cyclo (L- Phe-L-Pro (**1.40**) (Table S2) from *Vibrio vulnificus *modulates the expression of genes (ToxR) that are involved in the pathogenicity of *Vibrio spp *(91).

QS-mediated infections are most common with device implantations. Hence, Naresh Kumar and co-workers, 2013 made an attempt to curb the QS-mediated virulence in the implantation devices. They have covalently attached dihydropyrrolones (DHPs) to the glass surfaces and observed that such assemblies result in reduced attachment of the *P. aeruginosa *and *S. aureus* (**1.41-1.45**, Table S2) (92). 

A library of known QS inhibitors and related analogs of AHLs have been screened in order to find QS inhibitors against *Pectobacterium* that utilizes 3-oxo-N-octanoyl-homoserine lactone mediated QS to infect the potato plants and tubers. This screening has resulted in the identification of four *N*,*N’*-bisalkylated imidazolium salts that have shown QS inhibition in *Pectobacterium* based reporter organisms (93).

The research group of Zhao *et al. *in 2013 have synthesized *N*-sulfonyl homoserine lactone with *ortho *substituent on the phenyl ring (**1.46, **Table S2), effective in inhibiting the violacein production in *C. violaceum *and could be a good source of QS inhibitor with further structural modifications (79).

In an attempt for QS inhibitors, the MvfR (PqsR) mediated QS of *P. aeruginosa *was targeted using compounds with benzamide-benzimidazole scaffolds. This was found that these compounds have great therapeutic potential since they are not posing any effect on the growth of the persisters while targeting virulence. The most potent compound (**1.47**) (Table S2) possesses a nitro substitution on benzimidazole ring and phenoxy substitution at benzamide ring has significantly reduced the autoinducers (PQS and HHQ) and virulence factor (pyocyanin) production (94).

Further, in search of QS inhibitors, indole-based compounds were also analyzed. The natural compound oroidin having a trifluoromethoxy (OCF3) substitution (**1.48**) is potentially found to inhibit the biofilm formation of *S. mutans *and *S. aureus *(95). Kumar and co-workers reported the potential of the indole-based derivatives of the signaling molecule for QS inhibition. They have checked the *in-vitro *QS inhibitory activity using LasR receptor-based strain *plasB-gfp *(ASV). Among the library of compounds evaluated, one of the compound N-(2-phenyl-1H-indol-3-yl) hexanamide (**1.49**) (Table S2) was found to be most potent to act as QS inhibitor exhibiting more than 50% reduction in green fluorescent protein (GFP) production in *P. aeruginosa *(96).

In the year 2014, the research group of Hartmann has targeted the enzyme PqsD (*Pseudomonas* QSD), which is involved in the synthesis of autoinducer signal molecules in *P. aeruginosa. *In one of their reports, they showed QS’s inhibitory potential of benzamidobenzoic acids (**1.50**) and their derivatives as PqsD inhibitors, where they found that the chloro-substituted compounds showing promising PqsD inhibitory activity (97). In continuation, this research group also come up with another report showing 2- nitrophenyl methanol (**1.51**, Table S2) and its analogs as inhibitors of the PqsD enzyme (98).

Singh and co-workers have designed *N, N* disubstituted biguanides and synthesized its various analogs under microwave irradiation. The biological evaluation has shown that *N, N *disubstituted biguanides (**1.52**) framework possess QS inhibition activity against *C. violaceum* with IC_50 _ranging between 179 and 120 µM for its several derivatives (99).

In another attempt, our research group established the QS inhibition potential of novel unsymmetrical azines (1.53) and found that these compounds inhibit the QS-mediated GFP signals in a dose-dependent manner. Two active compounds also exhibited biofilm clearance at 50 μM concentration in *P. aeruginosa *(100).

*QS inhibitors of natural origin*

The natural product diversity holds immense potential for the occurrence of bioactive metabolites. A large number of bioactive have been isolated from microbes (101) with a diverse structural framework. 

A halogenated furanone compound having antifouling properties has been isolated from the red alga *D. pulchra *(102). In 1996 and later in 1999, Givskov *et al. *and Manefield *et al. *found the naturally occurring QS inhibitors by experimentally demonstrated that marine alga *D. pulchra *produces various halogenated furanones. These compounds interfere with the AHL-mediated QS deployed by the bacteria and thereby prevent surface colonization (103, 104). These furanones were found to inhibit QS of *P. aeruginosa, *where they reduced the *lasB-gfp *expressions, virulence factors production, and detaching the bacterial biofilm from the substratum by changing its structural framework (105). The mechanism by which natural furanone ((*5Z*)-4-bromo-5-(bromomethylene)-3-butyl-2(*5H*)-furanone) (**1.29**) inhibits the QS in *V. harveyi *have been exploited. This molecule prevents the binding of the transcriptional activator LuxR to the promoter sequences of QS-regulated genes (106).

**Figure 7:**
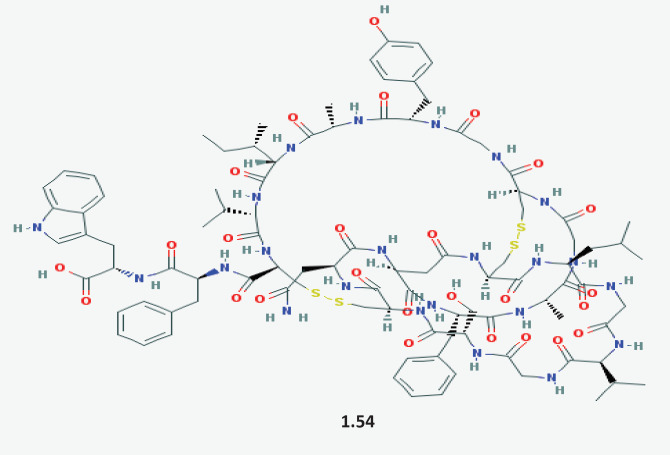
Structural representation of Siamycin

The peptide-based antibiotic siamycin (**1.54**) (Figure 7) from the supernatants of *Streptomyces sp. *culture (strainY33-1) was found to possess QS inhibition activity (107). Further, the TCM library has been screened for the presence of QS inhibition activity and the compound baicalein (**1.55**, Table S3, in supplementary file) has been found to show QS inhibition activity as it effectively reduced the biofilm formation (108).

Exploration of marine microbial diversity for screening of QS inhibitory activity is also reported in the literature. Various marine organisms were screened for the presence of QS inhibitory activity against *P. aeruginosa. *In this quest, they have isolated various sesquiterpenes metabolites like secomanoalide (**1.56**), manoalide (**1.57**) and manoalide monoacetate (**1.58**) from a sponge *Luffariella variabilis (L. variabilis) *(109) summarized in Table S3. All these molecules were found to be active against QS Inhibition Screen 1 (QSIS1) based on LuxR and QS Inhibition Screen 2 (QSIS2) based on LasR, respectively.

QS inhibition activity of essential oils against *C. violaceum *and *P. aeruginosa *have also been reported in the literature (110). Out of the various oils tested, clove oil along with others like cinnamon, lavender and peppermint oils were able to show QS inhibition activity as checked against CV026 and PA01 strains for violacein production and swarming motility, respectively. The active molecule responsible for QS inhibition activity from clove oil is yet to be separated as the major component of clove oil. However, eugenol did not show any QS inhibition activity (111). In a similar attempt, the activity of about 29 essential oil compounds was checked and 22 compounds were found active against *C. violaceum *and *P. aeruginosa *(112). The QS inhibition activity of pigment extracted from a fungus *Auricularia auricular *was also analyzed. The pigment was found to successfully inhibit the violacein production in the CV026 strain of *Chromobacterium* (113).

Flavonoids are naturally active compounds. With this perspective, several flavonoids derivatives such as naringenin, kaempferol, quercetin and apigenin were tested against *P. aeruginosa. *Out of the several tested compounds, naringenin (**1.59**, Table S3) isolated from the Malagasy plant *Combretum* *albiflorum *was found to show a marked reduction in the expression of multiple QS genes like *lasI, lasR, rhlI, rhlR, lasA, lasB, phzA1 *and *rhlA *in *P. aeruginosa* (114). 

Screening a library of 78 compounds derived from marine organisms like sponges, algae, fungi, tunicates, cyanobacteria and terrestrial plants against CV017 strain was also well analyzed. Out of these 78 collections, hymenialdisine (**1.60**), demethoxy encecalin (**1.61)**, microcolins A (**1.62**) and B (**1.63**) and kojic acid (**1.64**) were found to be active against the LuxR-based receptor of *E. coli *pSB1075 (Table S3). The best among them was kojic acid, which has shown growth inhibition and decreased densities of microbial communities on glass slides (115).

The QS inhibition activity of cinnamic acid (**1.65**) along with some linear dipeptides like proline–glycine (**1.66**) and N-amido-α-proline (**1.67**) have been evaluated (Table S3). These molecules were isolated from the extract of sponge-associated with actinomycetes and belong to the genus *Streptomyces *(116).

Cembranoid diterpenes analogs (**1.68**) isolated from *Eunicea knighti *compromising of knightal and knightol acetate were found to show QS inhibition activity against *C. violaceum. *All known and unknown cembranoid diterpenes were found to show QS inhibition activity as inhibiting the production of biofilm in *P. aeruginosa, S. aureus *and *V. harveyi *(117).

The isolation of honaucin, which is chemically a butyrolactone linked to a 4-chlorocrotonic acid via ester linkage, has been reported in the literature. The different analogs of honaucins viz honaucin A (**1.69**), honaucin B (**1.70**) and honaucin C (**1.71**), along with a few more, were checked for QS inhibition activity (Table S3). These molecules have the potential to show QS inhibition activity in *V. harveyi *BB120. The source of these QS inhibitory molecules is the cyanobacterium *Leptolyngbya crossbyana (L. crossbyana)*. The synthetic analog of this naturally occurring molecule, honaucin A *i.e*., 4’-bromohonaucin, poses more potent QS inhibition activity as compared to its natural counterpart (118).

In another report, the isolation of six sesquiterpene lactones (SLs) belonging to the class goyazensolide and isogoyazensolide-type were reported. These SLs were isolated from the argentine herb *Centratherum punctatum (C. punctatum) *and found to show QS inhibition activity as checked against *P. aeruginosa *for biofilm formation and other virulence factors production like elastase activity. One of the six molecules that is 1-Oxo-3,10-epoxy-8-epoxymethacryloyloxy-15-hydroxygermacra-2,4,11(13)-trien-6,12-olide (**1.72**, Table S3) found to show inhibition of biofilm formation at 1.32 µM concentration (119).

A compound protoanemonin (4-meth-ylenebut-2-en-4-olide) (**1.73**, Table S3) found to have QS inhibitory activity as it was able to reduce the expression of genes and virulence factors responsible for QS in *P. aeruginosa. *This molecule is produced by *Pseudomonas sp*. B13 and *P. reinekei *MT1 (120).

Sulfur-containing molecule ajoene (**1.74**, Table S3) isolated from the garlic extract using bioassay-guided fractionation is known to have QS inhibition activity. The ajoene is found to inhibit a multitude of genes responsible for QS in *Pseudomonas *(121). The various experiments have shown the ability of ajoene to inhibit genes responsible for virulence factors, rhamnolipid production, C4 and C12-AHL autoinducers synthesis and biofilm formation. Ajoene was also found successful for QS inhibition activity in the *in-vivo *studies.

The marine alga was also checked for the presence of QS inhibition against biomonitor organism CV026. They have found that alga *Asparagopsis taxiformis (A. taxiformis) *showed both antibacterial and QS inhibition activity. The active fraction showing QS inhibition activity as indicated by the inhibition of violacein production with CV026 was presumed to be 2-dodecanoyloxyethanesulfonate (C14H27O5S) (**1.75**, Table S3) based on the Fourier transform mass spectrometry data (122).

QS inhibition activity was also found to be associated with crude extracts of a fruit *Kigelia africana *against *Chromobacterium *and *A. tumefaciens *(123). In 2013, a *Pseudomonas sp*. whose culture supernatant was found to inhibit *rhl *mediated QS traits like pyocyanin and rhamnolipid production in the wild type PA01 (124). Eukaryotic hormonal communication on bacterial QS as found in the plant compound hordenine and the human sexual hormone estrone and the related compounds estriol and estradiol effectively decreases the accumulation of signaling molecule AHL expression of QS regulating genes (125). The fruit of *Terminalia chebula *found to contain active ingredients belonging to the class of ellagic acid derivative, which has suppressed the expression of *lasIR* and *rhlIR* QS genes, decreasing the production of virulence factors and increasing the susceptibility of PA01 biofilms (126). Vasavi *et al. *shown the presence of QS inhibitory activity in various herbs belonging to the class *Syzygium cumini *and *Pimenta dioica *for the inhibition of violacein production in *C. violaceum *CV31532 (127). In the year 2014, this group identified the presence of flavonoids quercetin and quercetin-3-O-arabinoside using LC-MS in the leaves of *Centella asiatica (C. asiatica), *which is showing QS inhibition activity against different strains of *C. violaceum *(128)*. *In 2016, this group showed that the flavonoid containing fractions of the herb *C. asiatica *has the ability to inhibit QS in *C. violaceum *and *P. aeruginosa *(129). 

Recently, screening the supernatants of about 5389 isolates, a strain JM2 belonging to the genus *Pseudomonas *found inhibiting LasR-mediated elastase and protease production in the wild type *P. aeruginosa *strain PAO1 (130). Six compounds belonging to xanthones (**1.76**, Table S4, in supplementary file) and mangostanaxanthones were identified as QS inhibitors against *C. violaceum* (131). The active ingredient of cranberry, which is proanthocyanidin (**1.77**, Table S4), reduces the swarming motility and biofilm formation in *P. aeruginosa *(132). QS inhibition activity of polyphenols-rich extracts of water-lily *Nymphaea tetragona* has been reported against *C. violaceum *and *Staphylococcus typhimurium *(133). In addition to polyphenols, a marine bacterium *Vibrio alginolyticus *G16 producing a phenolic compound (**1.78**, Table S4) also found to possess QS inhibitory activity against *S. marcescens *as indicated by reduced biofilm formation and virulent factors production (134). Another alcoholic compound, phytol (**1.79**, Table S4) found to inhibit the biofilm formation of *P. aeruginosa *(135). QS inhibitory activity associated with cyclic depsipeptides (skyllamycins) (**1.80**, Table S4) has also been reported. This was done by screening 312 natural products for QS inhibition activity using an image-based 384-well high-throughput screening assay (136). 

**Screening methods used for the identification of QS inhibitors**

The various methods used for the screening of QS inhibitors are broadly divided into two categories viz. Biological Screening and Non-Biological Screening. The screening of QS inhibitors carried out using various biomonitor organisms is classified under the biological screening method. However, in contrast to the above method, there is also non-biological screening which includes a) the combinatorial approach, which involves synthesizing a library of compounds by mainly using the solid-phase synthesis of peptide synthesizer (137). It is considered as one of the effective ways to screen a large number of compounds against multiple targets, b)* in-silico *approach, this is one of the widely used methods followed in rational drug design-based studies. It is considered a cost-effective method used for screening a large number of databases through high-performance computing. The use of informatics techniques increases the rate of success in drug discovery programs (138).

*Screening from Biological Sources*

The screening of the QS inhibitors from biological sources mainly focuses on the diversity of microorganisms for the presence of a diverse class of compounds that can act as QS inhibitor molecules. In this type of screening, the major step is to obtain a large number of microorganisms involving bacteria, fungus, algae, etc. and then check them for the presence of QS inhibition activity. The need for biosensors was realized quite early because of the increasing variation in the QS signals produced by diverse organisms. 

The use of biomonitor organisms for screening is most commonly employed to identify QS inhibitors from natural products. The most widely used biomonitor strain for screening QS inhibitors is *C. violaceum *in which the production of characteristic purple pigment violacein is under the QS-control (139). In this wild-type strain, violacein is inducible by all the AHLs compounds evaluated with N-acyl side chains from C4 to C8 in length, with varying degrees of sensitivity. In this category, the compounds (**1.81**-**1.84**, Table S5) have been identified as antifouling agents. This group has screened 78 natural products from chemical libraries using a reporter strain *C. violaceum *CV017. Compound **1.84** is the most effective inhibitor found in this study which reduces the formation of microbial communities in marine ecology (115).

Another equally effective biomonitor strain for screening long-chain AHL is *A. tumefaciens *A136 and KYC6 (140,141). The reporter strain *A. tumefaciens A136* (pCF218) (pCF372) lacks the Ti plasmid and contains two plasmids, pCF218 and pCF372, encode the *traR* and *traI-lacZ* fusion genes, respectively. This strain contains the β-galactosidase gene driven by a *traI* promoter, allowing the expression of β-galactosidase to be regulated by the presence of QS signals (AHLs). In the presence of the substrate (X-Gal), β-galactosidase enzymatically cleaves X-Gal, which results in its conversion to a blue precipitate when active forms of AHLs are present where the accumulation of the blue precipitate can be easily detectable by naked eyes. Compound **1.85** which is naphtho-furan fused heterocyclic is being identified as QS inhibitor by the use of A136/KCY6 strain where A136 responds to the autoinducer signal produced by KYC6 (123). On similar lines, Halwani *et al. *reported the inhibition of biofilm in *P. aeruginosa* using a liposomal formulation of bismuth–thiol (**1.86**) and tobramycin. They have found that in the presence of such formulation, there is reduced autoinducer production by *P. aeruginosa* as monitored by using the reporter strain A136 (142). 

In contrast to above biosensors, another *P. aeruginosa* based system is Pl*asB-gfp*(ASV) that contains a P*lasB-gfp*(ASV) translational fusion together with the *lasR* gene placed under the control of P*lac *(143). In this strain, the green fluorescence protein-coding genes were cloned with QS genes and hence the extent of fluorescence is under the control of the extent of quorum sensing. The GFP-based strains were used for high throughput screening of QS blockers by using two AHL biosensors: *Pseudomonas putida (P. putida)* F117(pKR-C12) and *P. putida* F177(pAS-C8). This method is used for the identification of compound **1.87 **(144). Similarly, compounds **1.88-1.90** are identified by using different strains of *P. putida* (145, 146).

*Screening of QS inhibitors from Non-biological sources*

*Combinatorial library screening*

The combinatorial synthesis approach has been deployed to synthesize a library of about two lakh compounds (89). Their library is based on diverse, commercial compounds, which were combined with a combinatorial library of 23 core scaffolds along with the known biologically active compounds. On screening this library against the QS inhibitory activity, only two compounds- V-06-018 (**1.33**) and PD12 (**1.34**, Table 2) were found to be active against *P. aeruginosa. *This study illustrates a very low success rate of just 0.0001% finding only two active leads that are simply analogs of the signaling molecule 3-Oxo-C12-AHL. Following this approach, Reyes-Arellano *et al., *in 2012, synthesized several derivatives of 2-substituted imidazolines and checked their QS inhibition activity against *C. violaceum CV12472* for violacein production. They have found that one of the compounds (**1.91**) (Table 2) is showing enhanced QS inhibition activity at 100 µm concentrations (147). This further clearly adds to the drastically low success rate of the combinatorial library approach for QS inhibitor molecules.

*Computational design of QS inhibitor*

Bioinformatics protein structure study tools helped to develop a rational drug design approach involving the SBDD approach (147-154). SBDD has been applied for the screening of QS inhibitors that can act as drug-like candidates (99, 100). In this context, a molecular docking-based virtual screening approach is also utilized to identify potent inhibitors of target proteins (155). Gupta et al. also employed similarity search-based virtual screening studies to repurpose the natural products against antiangiogenic targets (156). 

In order to get qualitative insights into protein-ligand binding sites, the homology model of LuxR on the template of TraR was developed and mostly the residues responsible for interaction with cognate AHL and the DNA fragment in TraR were found to be conserved in LuxR. A series with the analog of N-acyl homoserine lactones were docked inside the active site of the 3D model of LuxR. This group has also carried out molecular docking studies with a series of 11 new analogs of N-acyl homoserine lactones in which the carboxamide bond was replaced by a sulfonamide moiety (78).

The ligand-based virtual screening approach, which compromises of molecular alignment tool with an iterative database screening, has been utilized to develop novel compounds that specifically inhibit N-acyl-homoserine lactone dependent bacterial communication in members of the *Burkholderia sp. *The compound (**1.87**, Table S6, in supplementary file) is able to inhibit the *cep *QS in *B. cenocepacia *without killing the bacterial cells (157). In an attempt to find a QS inhibitor molecule, Taha *et al. *have screened National Cancer Institute (NCI) database against the LasR receptor (158). They reported the finding of a compound containing mercury (Hg) using a five-point pharmacophore model that has shown an enhanced binding affinity against the LasR receptor and also confirmed its activity through *in-vitro *assays. The compound (**1.92**, Table S6) was found to show the inhibitory activity at nanomolar ranges as indicated by reducing the formation of virulence factors (pyocyanin and pyoverdin) of *P. aeruginosa*. To study the vital insights of the active site of LuxR, the research group of Estephane has carried out molecular docking analysis to study the binding interaction of N-acyl-3-amino-5H-furanone derivatives in the active site pocket of QS receptor proteins. They have chosen the LuxR homology model PDB code: 1L3L of *V. fischeri *which is based on the TraR receptor protein of *A. tumefaciens *(159).

Bhatia *et al.* also utilized a molecular docking approach to identify three polyphenolic scaffolds gingerol (**1.93**), shogaol (**1.94**) and isoxazoline derivative of gingerol obtained from a ginger rhizome as inhibitors of CviR and LasR QS target proteins (152).

Fifty-one Traditional Chinese Medicines (TCM) compounds with known antibacterial activity were docked using the sphere selection method in the DOCK package into the active site of the transcriptional activation factor TraR. *In-vitro *screening of eight high-scoring compounds subsequently showed that *P. aeruginosa *growth was effectively inhibited by baicalein (**1.95**, Table S6) while acting synergistically with ampicillin (160).

The receptor-based virtual screening of 1.7 million compounds obtained from 14 different commercial databases against the LuxP receptor protein (PDB ID: 1JX6) of *V. harveyi *was carried out (161)*. *In this quest, two compounds (**1.96**-**1.97**, Table S5, in supplementary file) showing QS inhibition activity *in-vitro *with IC50 values of 35 and 55 µM, respectively, were found. The key interactions shown by these compounds were with Ser79, Arg215, Thr266, and Arg310 and Tyr81, Trp82, Asn159, Ile211, Phe206, and Ser265 amino acid residues. The common interactions are due to the presence of the sulfone group of both these compounds showing interactions with the Arg215 and Arg310 present in the active site of the receptor. This sulfone moiety closely resembles the borate moiety of AI-2 (**1.03**), which is the natural ligand of the receptor LuxP.

Similarly, in the hunt for finding QS inhibitors using computational approaches, the virtual screening of 2,344 chemical compounds was conducted. These compounds were docked within the LuxR, TraR and low active site sequences. The compounds showing good binding affinities as indicated by their binding scores were further checked for QS inhibition potential using LuxR dependant QS system. They have found various leads, viz. tamoxifen, sertraline, pimethixene, terfenadine, fendiline and calmidazolium. Out of these compounds, calmidazolium (**1.98**, Table S6) was reported as the most potent non-AHL type inhibitor of LuxR dependant QS system (162).

Another example of ligand-based virtual screening studies by employing natural ligand furanone- C30 (**1.30**) and inhibitor NPO (4-Nitro- pyridine N-oxide, an inhibitor) (**1.39**) as structural templates to screen a database of compounds against LasR using automated docking program Molegro Virtual Docker (MVD) was performed to find out putative LasR inhibitors. The three compounds which have shown significant activity against the LasR were salicylic acid, nifuroxazide and chlorzoxazone and they have also shown inhibition of *rhl *and *pqs *QS systems of *P. aeruginosa. *The key interactions made with the amino acid residues by the natural ligand oxo-dodecyl homoserine lactone (OdDHL) (**1.05**), known inhibitors furanone-C30 (**1.30**), NPO (**1.39**), and new inhibitors from virtual screening salicylic acid (**1.99**), nifuroxazide (**1.100**), and chlorzoxazone (**1.101**) with LasR receptor are listed in Table S7, in supplementary file (163).

The receptor-based pharmacophore model was adopted by Skovstrup and his group, where they performed a virtual screening workflow combining pharmacophore and structure-based approaches in search of novel LasR ligands. Interestingly, they found the non-AHL type of antagonists (**1.02-1.04**) for the LasR receptor (164). The key interactions have been tabulated in Table S7. On similar lines, Michael Givskov and co-workers performed a receptor-based virtual screening approach in order to discover novel QS inhibitor candidates. Molecular docking was performed on 3,040 natural compounds and their analogs using LasR receptor protein. This study has resulted in a compound 5-imino-4,6-dihydro-3H-1,2,3-triazolo[5,4-d]pyrimidin-7-one (**1.105**, Table S7), which inhibit a number of LasR mediated virulence factors including protease IV, chitinase and pyoverdine synthetases, extracellular DNA release and elastase production and thereby elaborating virtual screening as a powerful tool for screening chemical diversity of molecules as QS inhibitors (165).

A research group led by Kim *et al. *adopted a strategy of developing inhibitors of TraR protein on the basis of ligand-receptor interactions, which they explored through molecular docking technique (166). They have designed the structural analogs of 3-oxo-octanoyl homoserine lactone (**1.07**) by replacing the carboxamide bond of this natural ligand of TraR receptor protein with a nicotinamide and a sulfonamide bond in the ordeal to design derivatives of N-nicotinyl-L-homoserine lactones or N-Sulfonyl-L-homoserine lactones using *in-silico *molecular modeling based software SYBYL packages and these designed analogs were thereby synthesized using the solid-phase organic synthesis (SPOS) method. The derivatives of N-nicotinyl-L-homoserine lactones and N-Sulfonyl-L-homoserine lactones were docked in the active site of TraR receptor protein using the FlexX module of SYBYL packages. They have divided all the analogs into three groups viz. A, B and C, depending upon the substitutions present on the signaling molecule 3-oxo-octanoyl homoserine lactone. Their Group A compounds (**1.106**-**1.108**) formed by replacing the side chain with an aromatic ring structure, while the Group B compounds (**1.109 **and **1.110**) were having polar groups on the aromatic ring and in the Group C compounds (**1.111-1.113), **the carbonyl group (C = O) was substituted by sulfide moiety (Table 3). The *in-vitro *QS inhibition activity of these analogs was checked using an assay system based on A136/KYC6, which confirms their QS inhibition activity and hence thereby establishing a relationship between *in-silico *modeling and *in-vitro *activity (MIC) (166). 

In order to provide information on different Lux-based QS target proteins of various pathogenic microbes, we listed their PDB Id codes along with the resolution (Å) in Table 4. This information will facilitate the researchers working on the design and development of QS inhibitors by employing a structure-based drug design approach.

**Recent Advancements on QS inhibitors obtained from different sources**

Design and identification of QS inhibitors to target antimicrobial resistance are becoming a strategic approach that can help give new life to known antibiotics. QS inhibitors can also be classified based on their origin, like molecules produces by microbes, natural product-based (mostly plant origin) and chemically synthesized. In this section, we have tried to summarize the most recently found lead molecules associated with QS inhibition activity on the basis of their original source.

*QS inhibitors of Microbial origin*

Several recent reports (2018-20) demonstrate the isolation of potent bioactive compounds associated with QS inhibition against various infectious agents with a major focus on *P. aeruginosa* virulence. Table 5 provides the list of compounds, their structure along with the reported bioassay for confirmation of their activity. 

*QS inhibitors obtained from Natural Products*

Natural products are a potential source of bioactive including QS inhibitors. Table 6 shows compounds that are recently being identified (2018-20) as QS inhibitors, their organism against which they have shown activity, along with the description of biological assay. 

*QS inhibitors identified through Drug repurposing*

The recent data obtained from literature published in 2018-20 have proven the potential of many known drugs as QS inhibitors which are results of studies performed on FDA-approved drugs for drug repurposing motives. Mostly the evaluated drugs were found to interfere with QS signaling of *P. aeruginosa* with its different QS targets, as indicated by the activity of the tested compound among *in-vitro* assays. The complete summary of recently known drugs found active for showing QS inhibition activity has been summarized in Table 7.

**Table 1:** AI produced by different bacterial species which are involved in the process of QS

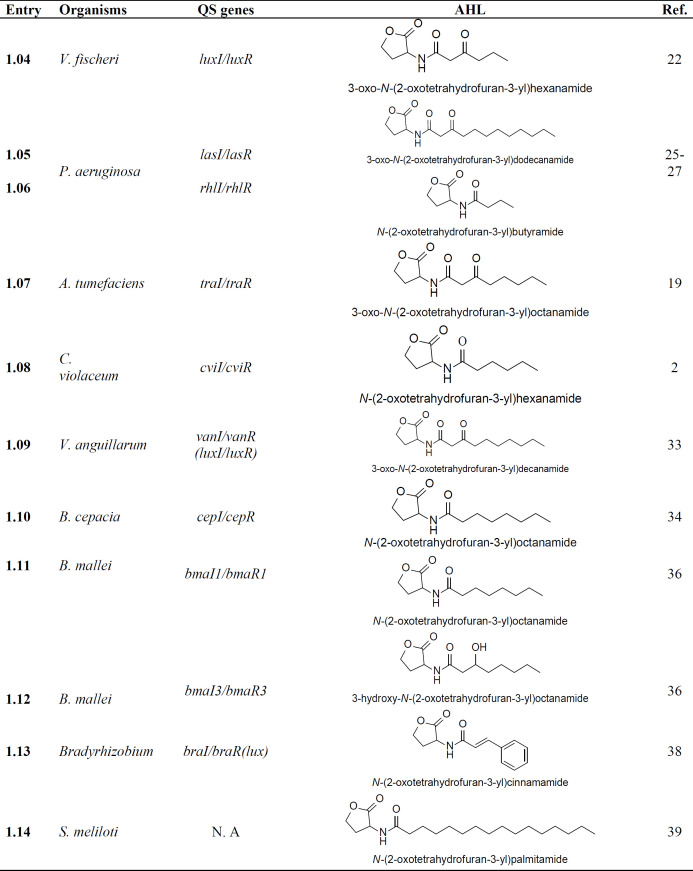

**Table 2:** List of molecules identified as QS inhibitors by the use of combinatorial screening approach

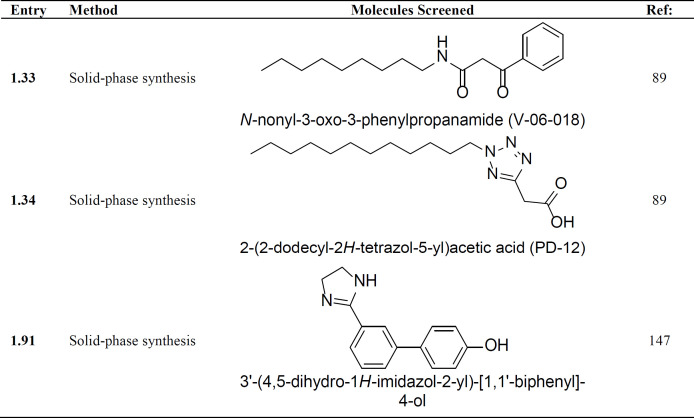

**Table 3:** Binding interactions are shown by molecules with TraR receptor

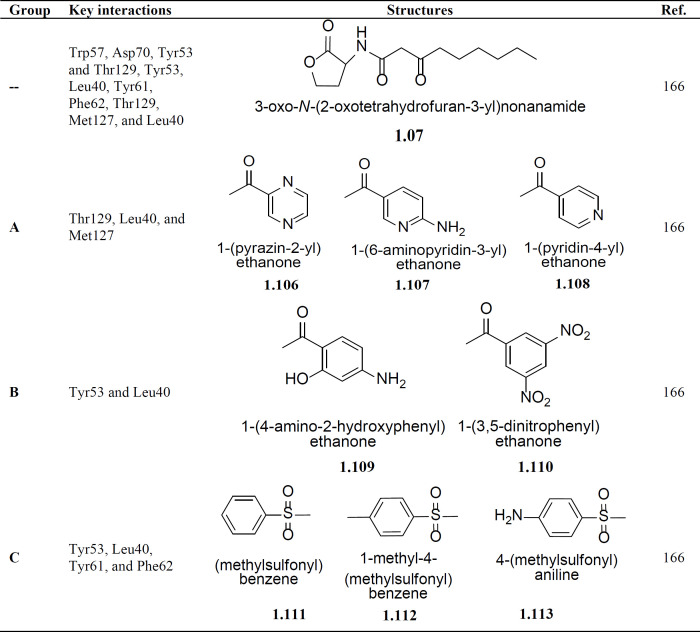

**Table 4:** List of PDB IDs of Lux based QS receptor proteins of different microbes

**Organism**	**PDB ID**	**Resolution (** **Å)**	**Ref.**
*C. violaceum*	3QP5 3QP6	3.25 2.00	
*P. aeruginosa*	2UV0 6MVN 6MVM	1.80 2.20 1.895	
*S. aureus*	3BSB1 4XY0 4XQ0	1.60 2.00 3.05	
*A. tumefaciens*	1H0M 1L3L	3.00 1.66	
*V. harveyi*	2HJ9 1ZHH	2.34 1.94	

**Table 5:** List of recently identified QS inhibitors from a microbial source

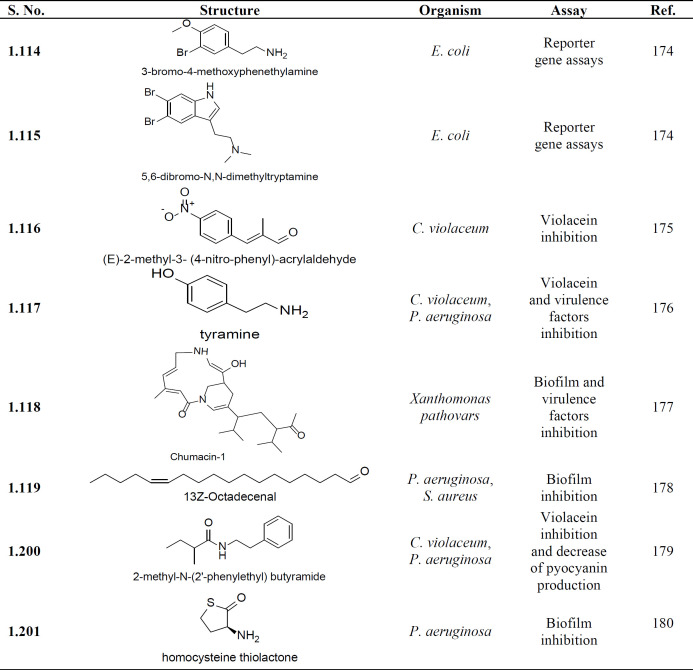

**Table 6:** List of recently identified QS inhibitors from natural plant and marine-based products

**S. No. **	**Compound name**	**Organism**	**Assay**	**Ref.**
**1.202**	Hordenine	*P. aeruginosa*	Biofilm and virulence factors inhibition	
**1.203**	Cinnamaldehyde	*P. fluorescens*	Biofilm and virulence factors inhibition	
**1.204**	Parthenolide	*P. aeruginosa*	Biofilm inhibition	
**1.205**	Z-phytol	*Salmonella*	Biofilm inhibition	
**1.206**	Flavonoids	*E. coli*, *P. aeruginosa*	Biofilm inhibition	
**1.207**	Pyrogallol	*C. violaceum*	Suppression of autoinducer (C_6_-AHL) production	
**1.208**	Carnosic Acid and Carnosol	*S. aureus*	luciferase reporter strain	

**Table 7:** List of recently identified drugs as QS inhibitors

**S. No.**	**Know Drug**	**Current Use**	**QS inhibition activity**		**Ref.**
			**Organism**	**QS Target**	
**1.209**	Sitagliptin	Anti-diabetic	*P. aeruginosa*	LasR	
**1.210**	Tenoxicam	Analgesic and Antipyretic	*P. aeruginosa*	LasR	
**1.211**	Pimozide	Antipsychotic	*P. aeruginosa*	PqSR	190.
**1.212**	Secnidazole	Antibiotic	*P. aeruginosa*	LasR	
**1.213**	Clofoctol	Antibiotic	*P. aeruginosa*	PqSR	
**1.214**	Meloxicam	Analgesic	*P. aeruginosa*	LasR	
**1.215**	Albendazole	Anti-parasitic	*C. violaceum* *P. aeruginosa*	CviR LasR	

## Conclusion

Enormous work carried out in the field of QS confirms the cell-cell communication among pathogenic bacterial species. Due to QS-mediated virulence, pathogens have developed drug resistance and there is a dire need to develop potent QS inhibitors. In this context, several approaches which involve the identification of QS inhibitors from microbial, natural and synthetic sources were adopted, which resulted in the identification of structural leads having quorum quenching capability. 

Drug repurposing is also a sensible strategy that leads to the identification of new therapeutic use for approved or investigational drugs and can be utilized in a systematic manner to find out putative QS inhibitors with minimal effort. The structure-based drug design approach is also exploited equally to identify structural analogs which can inhibit the QS system of *C. violaceum, P. aeruginosa, S. aureus, V. harveyi and A. tumefaciens* *etc*., which majorly display their phenotypic characteristics under the control of QS. In this category, albendazole, meloxicam, sitagliptin, etc. In a nutshell, it is possible to utilize the leads from all the resources like natural product diversity of plants and microbes, chemically synthesized compounds and the existing FDA-approved drugs to move towards a molecule that can solve the riddle for QS inhibitors.

## Supplementary Materials


