# Supplementary material for: Quorum Sensing Inhibitors: Curbing Pathogenic Infections through Inhibition of Bacterial Communication
Source: Iran J Pharm Res. 2021 Spring;20(2):486–514. doi: 10.22037/ijpr.2020.113470.14318 (PMC8457738; doi:10.22037/ijpr.2020.113470.14318)
Supplement: Supplementary file 1 [file ijpr-20-486-s001.pdf]

Supplementary Materials for

Quorum Sensing Inhibitors: Curbing Pathogenic Infections through Inhibition of Bacterial Communication

Shaminder Singh* and Sonam Bhatia

*To whom correspondence should be addressed. E-mail: shaminder2@gmail.com

Volume 20, Issue 2 (Spring 2021)

This PDF file includes:
Tables S1-S7

Table S1. AHL based QS inhibitors.

**AHL head group containing analogues**		
**Entry**	**Structure**	**Ref.**
**1.17**	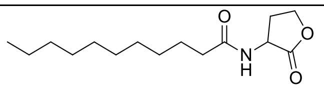 *N*-(2-oxotetrahydrofuran-3-yl)undecanamide	74
**1.18**	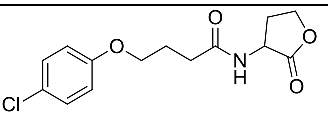 4-(4-chlorophenoxy)-*N*-(2-oxotetrahydrofuran-3-yl)butanamide	74
**1.19**	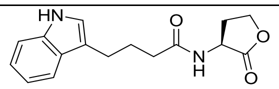 (*S*)-4-(1*H*-indol-3-yl)-*N*-(2-oxotetrahydrofuran-3-yl)butanamide	75
**1.20**	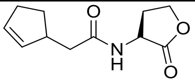 2-(cyclopent-2-en-1-yl)-*N*-((*R*)-2-oxotetrahydrofuran-3-yl)acetamide	75
**1.21**	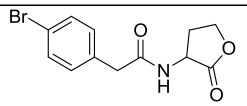 2-(4-bromophenyl)-*N*-(2-oxotetrahydrofuran-3-yl)acetamide	76
**1.22**	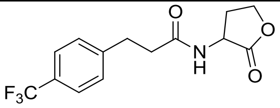 *N*-(2-oxotetrahydrofuran-3-yl)-3-(4-(trifluoromethyl)phenyl)propanamide	76
**1.23**	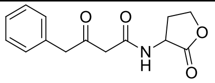 3-oxo-*N*-(2-oxotetrahydrofuran-3-yl)-4-phenylbutanamide	77
**1.24**	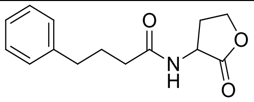 *N*-(2-oxotetrahydrofuran-3-yl)-4-phenylbutanamide	77
**1.25**	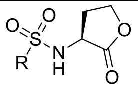 Sulfonamides	78
**Non-AHL Head group containing analogs**		
**1.26**	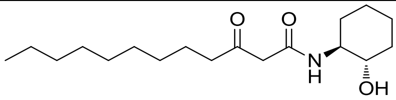 *N*-((1*S*,2*S*)-2-hydroxycyclohexyl)-3-oxododecanamide	81
**1.27**	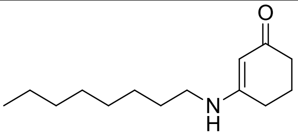 3-(octylamino)cyclohex-2-enone	82

**1.28**	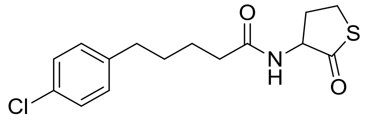 5-(4-chlorophenyl)-*N*-(2-oxotetrahydrothiophen-3-yl)pentanamide	83
**1.29**	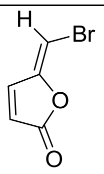 (*Z*)-5-(bromomethylene)furan-2(5*H*)-one	85
**1.30**	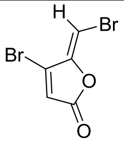 (*Z*)-4-bromo-5-(bromomethylene) furan-2(5*H*)-one	85
**1.31**	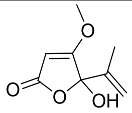 5-hydroxy-4-methoxy-5-(prop-1-en-2-yl)furan-2(5*H*)-one	87
**1.32**	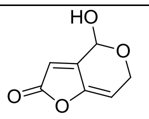 4-hydroxy-4,6-dihydro-2*H*-furo[3,2-*c*]pyran-2-one	87

Table S2. Structures of Non-AHL based QS inhibitor analogs.

Entry	Chemical class	Structure	Reference
**1.33**	Oxo-phenyl propenamide	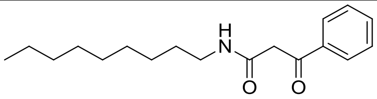 *N*-nonyl-3-oxo-3-phenylpropanamide	89
**1.34**	Tetrazole	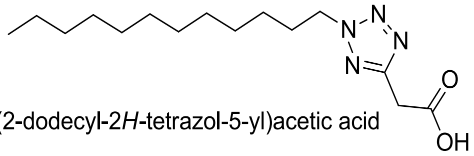 2-(2-dodecyl-2*H*-tetrazol-5-yl)acetic acid	89
**1.35**	Para-benzoquinone	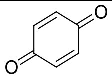 benzoquinone	90
**1.36**	Imidazole	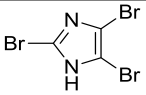 2,4,5-tribromo-1*H*-imidazole	90
**1.37**	Indole	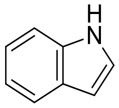 1*H*-indole	90
**1.38**	Sulfonamide	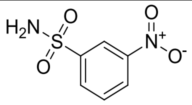 3-nitrobenzenesulfonamide	90
**1.39**	Pyridine-N-oxide	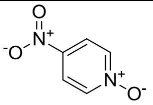 4-nitropyridine 1-oxide	90
**1.40**	Dipeptides	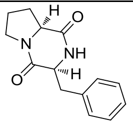 (3*S*,8a*S*)-3-benzylhexahydropyrrolo [1,2-*a*]pyrazine-1,4-dione	91
**1.41**	Dihydropyrrolones	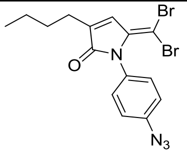 1-(4-azidophenyl)-3-butyl-5-(dibromomethylene)-1*H*-pyrrol-2(5*H*)-one	92

**1.42**	--do--	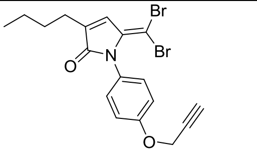<br>3-butyl-5-(dibromomethylene)-1-(4-(prop-2-yn-1-yloxy)phenyl)-1*H*-pyrrol-2(5*H*)-one	92
**1.43**	--do--	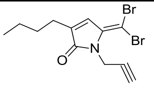<br>3-butyl-5-(dibromomethylene)-1-(prop-2-yn-1-yl)-1*H*-pyrrol-2(5*H*)-one	92
**1.44**	--do--	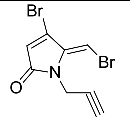<br>(*Z*)-4-bromo-5-(bromomethylene)-1-(prop-2-yn-1-yl)-1*H*-pyrrol-2(5*H*)-one	92
**1.45**	--do--	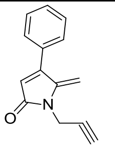<br>5-methylene-4-phenyl-1-(prop-2-yn-1-yl)-1*H*-pyrrol-2(5*H*)-one	92
**1.46**	Benzamide	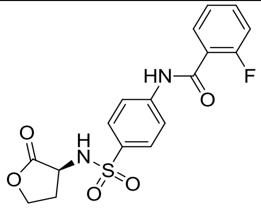<br>(*S*)-2-fluoro-*N*-(4-(*N*-(2-oxotetrahydrofuran-3-yl) sulfamoyl)phenyl)benzamide	79
**1.47**	Benzamide-benimidazole	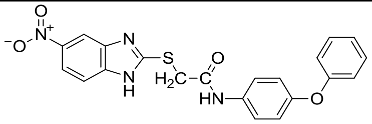<br>2-((5-nitro-1*H*-benzo[*d*]imidazol-2-yl)thio)-*N*-(4-phenoxyphenyl)acetamide	94
**1.48**	Indole	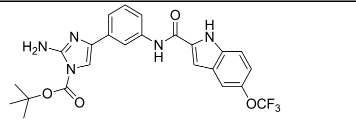<br>*tert*-butyl 2-amino-4-(3-(5-(trifluoromethoxy)-1*H*-indole-2-carboxamido)phenyl)-1*H*-imidazole-1-carboxylate	95
**1.49**	Indole	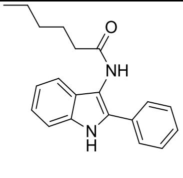<br>*N*-(2-phenyl-1*H*-indol-3-yl)hexanamide	96
**1.50**	Benzamido-benzoic acid	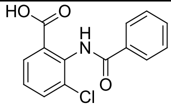<br>2-benzamido-3-chlorobenzoic acid	97

**1.51**	Nitro-phenyl methanol	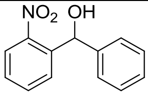	98
**1.52**	Biguanides	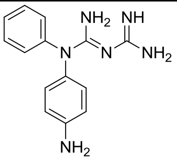	99
**1.53**	Azines	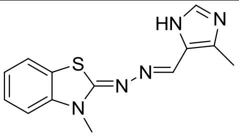	100

Table S3. Various leads identified from the screening of natural resources for QS inhibition activity.

Entry	Source	Bioassay strain	Assay method	Structure	Ref.
**1.29**	*D. pulchra*	*P. aeruginosa*	Biofilm inhibition assay	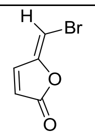<br>(*Z*)-5-(bromomethylene)furan-2(5*H*)-one	106
**1.55**	*Streptomyces albus A66*	*Vibrio spp.*	Biofilm inhibition assay	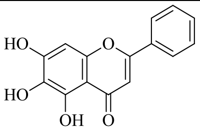<br>5,6,7-trihydroxy-2-phenyl-4*H*-chromen-4-one	108
**1.56**	*L. variabilis*	*P. aeruginosa*	P*lasB-gfp* (ASV) Bioassay	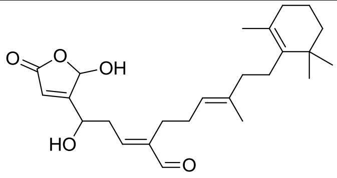<br>(2*E*,5*E*)-2-(3-hydroxy-3-(2-hydroxy-5-oxo-2,5-dihydrofuran-3-yl) propylidene)-6-methyl-8-(2,6,6-trimethylcyclohex-1-en-1-yl)oct-5-enal	109
**1.57**	*L. variabilis*	*P. aeruginosa*	P*lasB-gfp* (ASV) Bioassay	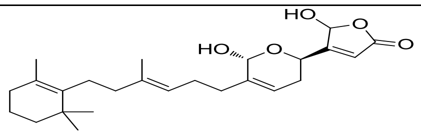<br>5-hydroxy-4-((2*R*,6*R*)-6-hydroxy-5-((*E*)-4-methyl-6-(2,6,6-trimethylcyclohex-1-en-1-yl)hex-3-en-1-yl)-3,6-dihydro-2*H*-pyran-2-yl)furan-2(5*H*)-one	109
**1.58**	*L. variabilis*	*P. aeruginosa*	P*lasB-gfp* (ASV) Bioassay	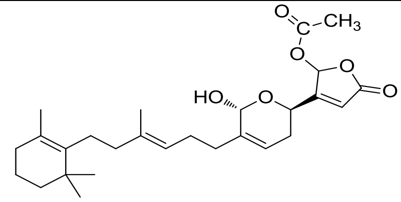<br>3-((2*R*,6*R*)-6-hydroxy-5-((*E*)-4-methyl-6-(2,6,6-trimethylcyclohex-1-en-1-yl)hex-3-en-1-yl)-3,6-dihydro-2*H*-pyran-2-yl)-5-oxo-2,5-dihydrofuran-2-yl acetate	109
**1.59**	*Combretum albiflorum*	*P. aeruginosa*	Virulence factor quantification	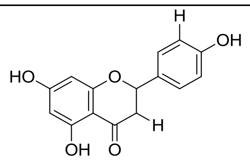<br>5,7-dihydroxy-2-(4-hydroxyphenyl)chroman-4-one	131
**1.60**	*Hymeniacidon aldis*	*C. violaceum CV017*<br>*V. fischeri*	Agar diffusion assay Bioluminescence assay	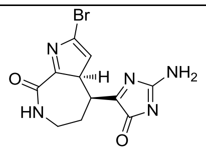<br>(3a*R*,4*S*)-4-(2-amino-4-oxo-4*H*-imidazol-5-yl)-2-bromo-4,5,6,7-tetrahydropyrrolo[2,3-*c*]azepin-8(3a*H*)-one	132
**1.61**	*Baccharis cassinaefolia*	*C. violaceum CV017*<br>*V. fischeri*	Agar diffusion assay Bioluminescence assay	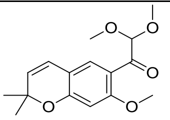<br>2,2-dimethoxy-1-(7-methoxy-2,2-dimethyl-2*H*-chromen-6-yl)ethanone	132

**1.62**	*Lyngbya sp.*	*C. violaceum CV017*<br>*V. fischeri*	Agar diffusion assay Bioluminescence assay	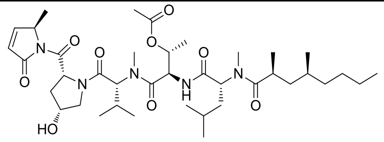<br>(2*R*,3*R*)-4-(((*R*)-1-((2*R*,4*R*)-4-hydroxy-2-((*R*)-2-methyl-5-oxo-2,5-dihydro-1*H*-pyrrole-1-carbonyl) pyrrolidin-1-yl)-3-methyl-1-oxobutan-2-yl)(methyl)amino)-3-((*R*)-4-methyl-2-((2*S*,4*S*)-*N*,2,4-trimethyloctanamido)pentanamido)-4-oxobutan-2-yl acetate	132
**1.63**	*Lyngbya sp.*	*C. violaceum CV017*<br>*V. fischeri*	Agar diffusion assay Bioluminescence assay	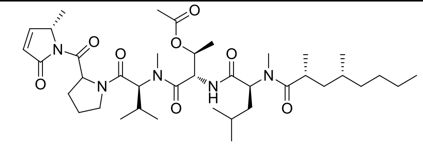<br>(2*S*,3*S*)-4-(methyl((2*S*)-3-methyl-1-(2-((*S*)-2-methyl-5-oxo-2,5-dihydro-1*H*-pyrrole-1-carbonyl) pyrrolidin-1-yl)-1-oxobutan-2-yl)amino)-3-((*S*)-4-methyl-2-((2*R*,4*R*)-*N*,2,4-trimethyloctanamido) pentanamido)-4-oxobutan-2-yl acetate	132
**1.64**	*Aspergillus spp.*	*C. violaceum CV017*<br>*V. fischeri*	Agar diffusion assay Bioluminescence assay	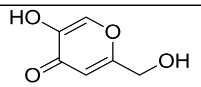<br>5-hydroxy-2-(hydroxymethyl)-4*H*-pyran-4-one	132
**1.65**	*Streptomyces* sp.	*C. violaceum* CV12472 *P. aeruginosa* ATCC 27853	Violacein inhibition assay, Virulence factors and biofilm inhibition	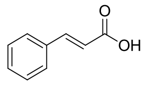<br>cinnamic acid	132
**1.66**	*Streptomyces* sp.	*C. violaceum* CV12472<br>*P. aeruginosa* ATCC 27853	Violacein inhibition assay, Virulence factors and biofilm inhibition	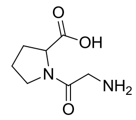<br>1-(2-aminoacetyl)pyrrolidine-2-carboxylic acid	133
**1.67**	*Streptomyces* sp.	*C. violaceum* CV12472<br>*P. aeruginosa* ATCC 27853	Violacein inhibition assay, Virulence factors and biofilm inhibition	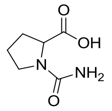<br>1-carbamoylpyrrolidine-2-carboxylic acid	133
**1.68**	*Eunicea knighti*	*P. aeruginosa, S. aureus, V. harveyi*	Biofilm inhibition	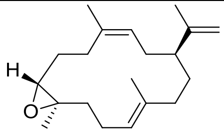<br>(4*E*,8*S*,10*Z*,14*R*)-1,5,11-trimethyl-8-(prop-1-en-2-yl)-15-oxabicyclo[12.1.0]pentadeca-4,10-diene	134
**1.69**	*L. crossbyana*	*V. harveyi* BB120	Bioluminescence assay	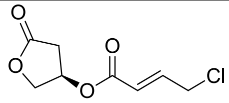<br>(*R*,*E*)-5-oxotetrahydrofuran-3-yl 4-chlorobut-2-enoate	135
**1.70**	*L. crossbyana*	*V. harveyi* BB120	Bioluminescence assay	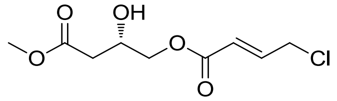<br>(*S*,*E*)-4-methoxy-2-hydroxy-4-oxobutyl 4-chlorobut-2-enoate	135
**1.71**	*L. crossbyana*	*V. harveyi* BB120	Bioluminescence assay	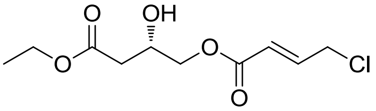<br>(*S*,*E*)-4-ethoxy-2-hydroxy-4-oxobutyl 4-chlorobut-2-enoate	135

**1.72**	*C. punctatum*	*P. aeruginosa*	Virulence factors and biofilm inhibition	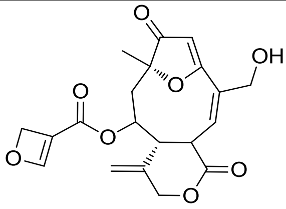 (4a*R*,7*S*,*Z*)-11-(hydroxymethyl)-7-methyl-4-methylene-1 ,8-dioxo-3,4,4a,5,6,7,8,12a-octahydro-1*H*-7,10-epoxycyclodeca[*c*]pyran-5-yl 2*H*-oxete-3-carboxylate	136
**1.73**	*Pseudomonas sp*. B13 and *Pseudomonas reinekei* MT1	*P. aeruginosa*	Virulence factors and biofilm inhibition	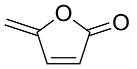 5-methylenefuran-2(5*H*)-one	137
**1.74**	*Allium sativum*	*P. aeruginosa*	Virulence factors and biofilm inhibition	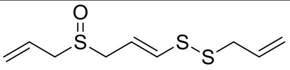 (*E*)-1-allyl-2-(3-(allylsulfinyl) prop-1-en-1-yl)disulfane	138
**1.75**	*A. taxiformis*	*Serratia liquefaciens*	*S. liquefaciens* MG44 bioassay	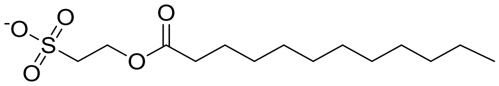 2-(dodecanoyloxy)ethanesulfonate	139

Table S4. List of various QS inhibitor molecules from natural sources.

Entry	Source	Bioassay strain	Assay method	Structure	Ref.
**1.76**	*Garcinia mangostan*	*C. violaceum* ATCC 12472	Agar diffusion assay	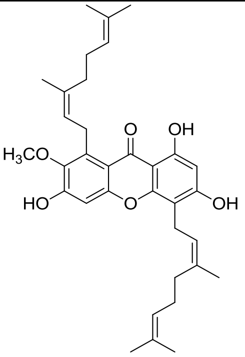 1,5-bis((*Z*)-3,7-dimethylocta-2,6-dien-1-yl)-3,6,8-trihydroxy-2-methoxy-9*H*-xanthen-9-one	131
**1.77**	Cranberry	*P. aeruginosa PAO1*	Biofilm inhibition assay	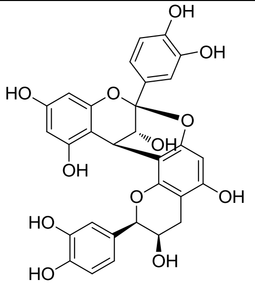 (2*R*,3*R*,8*S*,14*R*,15*R*)-2,8-bis(3,4-dihydroxyphenyl) -2,3,4,14-tetrahydro-8,14-methanobenzo [7,8][1,3]dioxocino[4,5-*h*]chromene-3,5,11,13,15-pentaol	132
**1.78**	*Vibrio alginolyticus* G16	*S. marcescens*	*S. marcescens* virulence assays	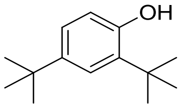 2,4-di-*tert*-butylphenol	134
**1.79**	Green vegetables	*P. aeruginosa* PAO1	Biofilm inhibition Assay	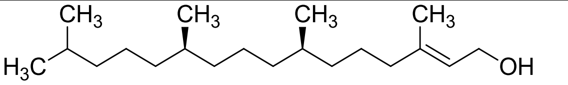 (7*R*,11*R*,*E*)-3,7,11,15-tetramethylhexadec-2-en-1-ol	135
**1.80**	*Streptomyces* sp. strain 1675	*P. aeruginosa* PAO1	Biofilm inhibition assay using HTS	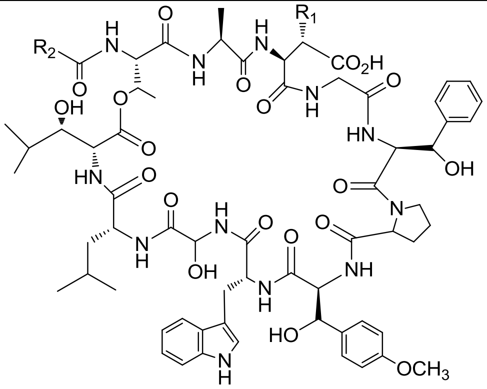 Cylic desipeptide	136

Table S5. List of compounds identified as QS inhibitors by using a biological screening method.

**Entry**	**Biomonitor organism**	**Strain used**	**Compound isolated**	**Ref.**
**1.81**	*C. violaceum*	CV017	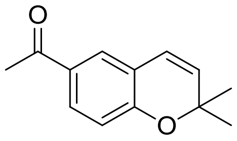 1-(2,2-dimethyl-2*H*-chromen-6-yl)ethanone	115
**1.82**	*C. violaceum*	CV017	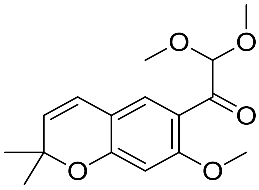 2,2-dimethoxy-1-(7-methoxy-2,2-dimethyl-2*H*-chromen-6-yl)ethanone	115
**1.83**	*C. violaceum*	CV017	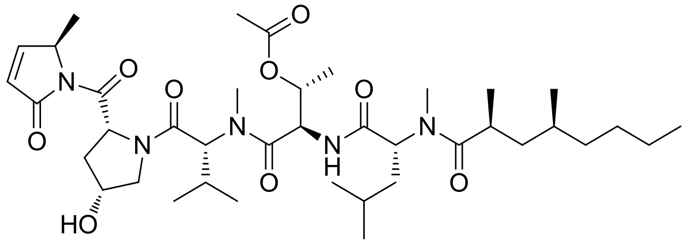 (2*R*,3*R*)-4-(((*R*)-1-((2*R*,4*R*)-4-hydroxy-2-((*R*)-2-methyl-5-oxo-2,5-dihydro-1*H*-pyrrole-1-carbonyl) pyrrolidin-1-yl)-3-methyl-1-oxobutan-2-yl)(methyl)amino)-3-((*R*)-4-methyl-2-((2*S*,4*S*)-*N*,2,4-trimethyloctanamido)pentanamido)-4-oxobutan-2-yl acetate	115
**1.84**	*C. violaceum*	CV017	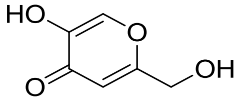 5-hydroxy-2-(hydroxymethyl)-4*H*-pyran-4-one	115
**1.85**	*A. tumefaciens*	A136/KYC6	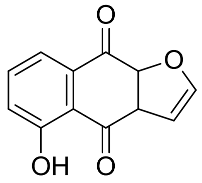 5-hydroxynaphtho[2,3-*b*]furan-4,9(3a*H*,9a*H*)-dione	123
**1.86**	*A. tumefaciens*	A136	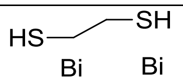 bismuth–ethanedithiol	142

**1.87**	*P. putida*	117(pAS-C8)-CepR receptor	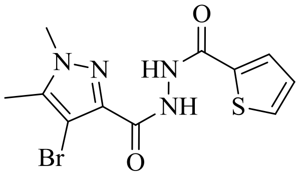 4-bromo-1,5-dimethyl-*N*'-(thiophene-2-carbonyl)-1*H*-pyrazole-3-carbohydrazide	144
**1.88**	*P. putida*	IsoF/gfp	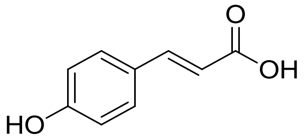 4-hydroxycinnamic acid	145
**1.89**	*P. putida*	F117(pKRC12)	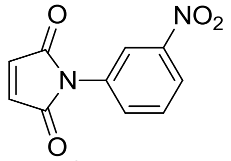 1-(3-nitrophenyl)-1*H*-pyrrole-2,5-dione	146
**1.90**	*P. putida*	F117(pKRC12)	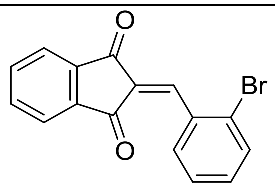 2-(2-bromobenzylidene)-1*H*-indene-1,3(2*H*)-dione	146

Table S6. List of compounds identified as QS inhibitors by the use of *in silico* techniques

Entry	*In-silico* technique	Method	Structure identified	Microorganism	Verification Assay	Ref.
**1.87**	Docking based Virtual screening	Ligand-based Virtual screening	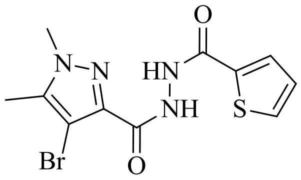 4-bromo-1,5-dimethyl-*N*'-(thiophene-2-carbonyl)-1*H*-pyrazole-3-carbohydrazide	*B. cenocepacia*	Proteolytic activity	157
**1.92**	Pharmacophore mapping	Ligand-based pharmacophore	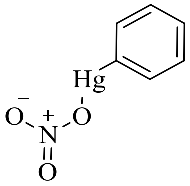 (nitrooxy)(ph enyl)mercury	*P. aeruginosa*	Virulence factor inhibition	158
**1.93**	Molecular docking	Broyden-Fletcher-Goldfarb-Shanno algorithm	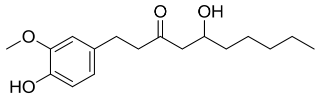 [6]-Gingerol	*P. aeruginosa*	Biofilm assay	152
**1.94**	Molecular docking	Broyden-Fletcher-Goldfarb-Shanno algorithm	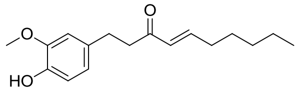 [6]-Shogaol	*P. aeruginosa*	Biofilm assay	152
**1.95**	Molecular docking	Anchor-and-grow *algorithm*	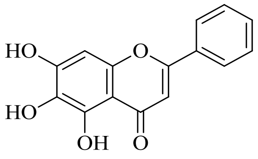 5,6,7-trihydroxy-2-phenyl-4*H*-chromen-4-one	*P. aeruginosa*	Biofilm inhibition	169
**1.96**	Docking based Virtual screening	Anchor-and-grow *algorithm*	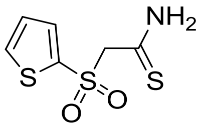 2-(thiophen-2-ylsulfonyl) ethanethioamide	*V. harveyi*	Bioluminescence assay	170
**1.97**	Docking based Virtual screening	Anchor-and-grow *algorithm*	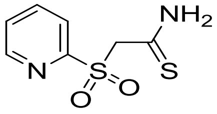 2-(pyridin-2-ylsulfonyl) ethanethioamide	*V. harveyi*	Bioluminescence assay	170

**1.98**	Docking based Virtual screening	Genetic algorithm	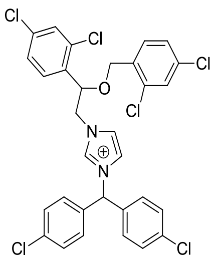 3-(bis(4-chlorophenyl)methyl)-1-(2-((2,4-dichlorobenzyl)oxy)-2-(2,4-dichlorophenyl)ethyl)-1*H*-imidazol-3-ium	*V. fischeri*	Bioluminescence assay	171

Table S7. Binding interactions shown by various ligands in the active site pocket of LasR receptor.

Entry	Compounds	Key interactions	Structure	Ref.
**1.05**	OdDHL	Trp60, Tyr56, Asp73, Ser129, Thr75	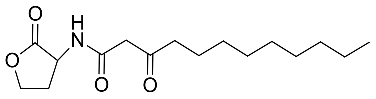 3-oxo-*N*-(2-oxotetrahydrofuran-3-yl)dodecanamide	51
**1.39**	NPO	Trp60, Tyr93	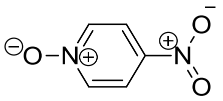 4-nitropyridine 1-oxide	51
**1.30**	Furanone-C30	Leu110	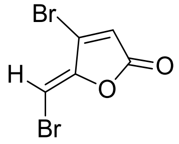 (*Z*)-4-bromo-5-(bromomethylene)furan-2(5*H*)-one	51
**1.99**	Salicylic acid	Trp-60, Tyr56, Asp73, Thr75, Ser 129	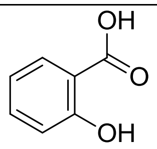 2-hydroxybenzoic acid	51
**1.100**	Nifuroxazide	Tyr56, Tyr64, Tyr93, Leu110, Asp73, Ser129 Tyr93	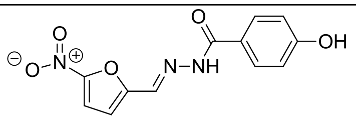 (*E*)-4-hydroxy-*N*'-((5-nitrofuran-2-yl)methylene)benzohydrazide	51
**1.101**	Chlorzoxazone	Tyr93	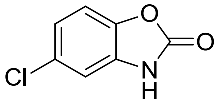 5-chlorobenzo[*d*]oxazol-2(3*H*)-one	51
**1.102**	Benzene-sulfonamide	Phe101, Tyr56, Trp60, Asp73	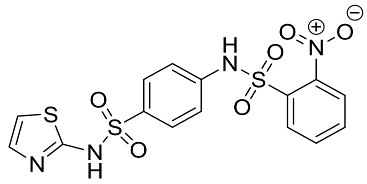 2-nitro-*N*-(4-(*N*-(thiazol-2-yl)sulfamoyl) phenyl)benzenesulfonamide	172

**1.103**	--do--	Phe101, Tyr56, Trp88 Asp73	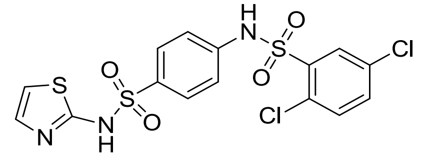<br>2,5-dichloro-*N*-(4-(*N*-(thiazol-2-yl)sulfamoyl) phenyl)benzenesulfonamide	172
**1.104**	-do--	Phe101, Tyr56, Trp88	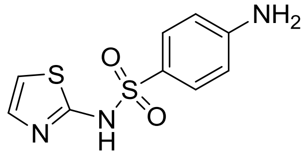<br>4-amino-*N*-(thiazol-2-yl) benzenesulfonamide	172
**1.105**	Triazolo-pyrimidin-one	Tyr93,<br>Thr75	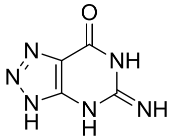<br>5-imino-5,6-dihydro-3*H*-[1,2,3]triazolo[4,5-*d*]pyrimidin-7(4*H*)-one	173
